# Laparoscopic Management of a Giant Simple Hepatic Cyst: A Case Report

**DOI:** 10.7759/cureus.101942

**Published:** 2026-01-20

**Authors:** Mohit K Badgurjar, Isha Purohit, Esha Nimawat, Sanjeev Agarwal, Yashpalsinh Rathod

**Affiliations:** 1 General Surgery, Geetanjali Medical College and Hospital, Udaipur, IND

**Keywords:** benign liver cyst, giant hepatic cyst, laparoscopic cyst deroofing, laparoscopic management, simple hepatic cyst

## Abstract

Simple hepatic cysts follow an indolent course and are discovered incidentally on imaging modalities, often requiring no treatment. However, for large hepatic cysts presenting with pressure symptoms, treatment becomes necessary.

We present a case of a 51-year-old woman presenting with complaints of progressive swelling over the right upper abdomen for one month, associated with abdominal pain for 15 days. She had undergone percutaneous aspiration for the same pathology previously done at another facility 15 days prior to presentation. Contrast-enhanced computed tomography (CT) revealed a 25x20x17 cm, well-defined, fluid-filled homogeneous cyst occupying the right lobe of the liver. Based on the general physical examination, elevated inflammatory markers such as C-reactive protein (CRP), and imaging findings, a diagnosis of an infected giant hepatic cyst was made. Laparoscopic cyst deroofing was planned. Intraoperatively, percutaneous aspiration of fluid, which yielded approximately 4 L of dirty fluid containing debris, was performed prior to cyst deroofing. The procedure was completed without any complications, and postoperative events were uneventful.

This case demonstrates that laparoscopic cyst deroofing can be successfully performed even for large cysts (25x20x17 cm). The key factors determining successful management include careful patient selection, cyst characters, and surgeon-related factors.

## Introduction

With an estimated prevalence of approximately 2.5% to 18% in the general population, simple hepatic cysts are common benign lesions found in the liver [1]. They are believed to originate from aberrant biliary ducts during embryogenesis and are lined by simple cuboidal epithelium of biliary origin [2]. Most hepatic cysts undergo an asymptomatic course and are discovered incidentally on imaging modalities such as ultrasound, CT, or MRI [3]. 

In the literature, cysts larger than 10 cm have been described as giant hepatic cysts [4]. These are relatively uncommon, occurring in 5% of the population [3]. Patients are typically asymptomatic; if symptomatic, they present with complaints of abdominal distention, nausea, vomiting, and pain, owing to the mass effect imparted on the adjacent organs [5]. In such patients, management options include percutaneous aspiration of fluid with sclerotherapy (PAS) and open or laparoscopic cyst deroofing [6]. Although open cyst deroofing has been preferred for giant hepatic cysts due to technical considerations such as limited working space and risk of bleeding and rupture, with recent advancements in minimally invasive surgery, the role of laparoscopic cyst deroofing has expanded in such cases [7]. In this report, we present a case of an infected giant hepatic cyst that was treated successfully using laparoscopic cyst deroofing, highlighting the role of minimally invasive surgery in such cases.

## Case presentation

A 51-year-old woman presented to the outpatient department of our institute with complaints of increasing swelling in her right upper abdomen for one month, pain in the epigastric region for 15 days, and intermittent fever for 10 days. The patient was initially treated at another center 15 days ago for the same complaint, where percutaneous aspiration of the cyst fluid was attempted. Following the procedure, she experienced worsening abdominal pain and fever, possibly due to superadded infection.

On history and general physical examination, the swelling was visible on the right side of the abdomen. The mass was soft in consistency, tender and moved with respiration. CT scan findings revealed a 25x20x17 cm, well-defined, fluid-filled homogeneous cyst occupying the right lobe of the liver. The cyst was present in segment VI, VII and VIII. Laboratory findings revealed an increased total leukocyte count (TLC) of 30,000/mm³ and a C-reactive protein level (CRP) of 50 mg/L. Other past medical history included history of hypertension since three years, managed with medication. Based on history and general physical examination, a provisional diagnosis of an infected giant hepatic cyst was made. In view of these findings and after discussion with the patient, laparoscopic cyst deroofing was planned.

**Figure 1:**
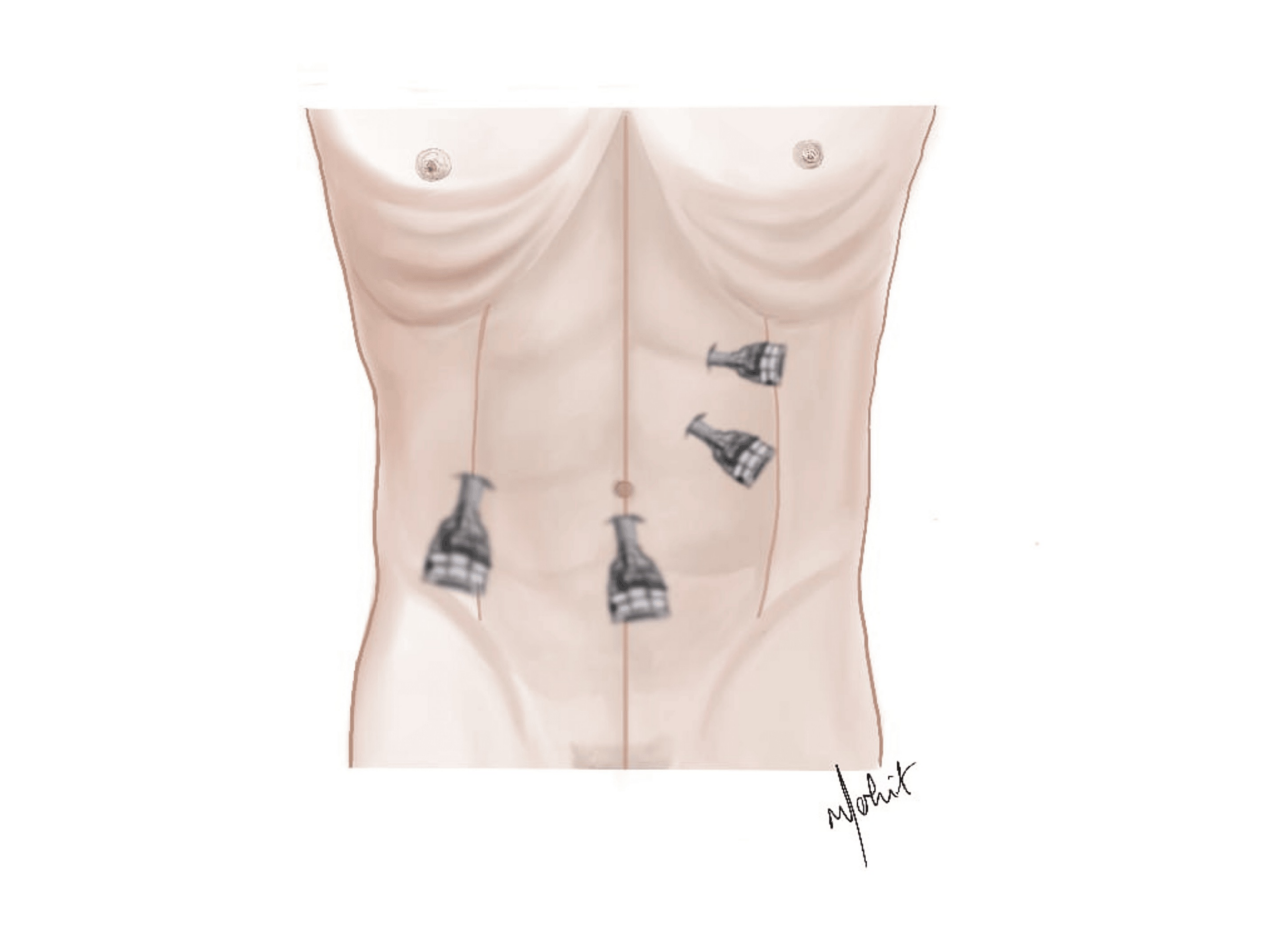
Schematic illustration of laparoscopic port placement showing a 10-mm infra-umbilical camera port, two 5-mm working ports in the right upper abdomen, and an additional 10-mm port for specimen retrieval and suction. Original illustration created using Procreate by Mohit K. Badgurjar. (Procreate is a licensed, one-time-purchase software developed by Savage Interactive Pty Ltd., Hobart, Tasmania, Australia.)

The patient was taken for surgery under general anesthesia. After pneumoperitoneum was established, port placement was done. A 10-mm camera port was inserted in the infra-umbilical region, and two 5-mm working ports were inserted in the right upper abdomen; an additional 10-mm port was placed to facilitate the retrieval of specimens and suction (as shown in Figure 1). Laparoscopic findings included one giant hepatic cyst arising from the right lobe of the liver. Initial decompression was performed by aspirating approximately 4 L of dirty fluid containing debris (suggestive of an infective cyst). Following aspiration, laparoscopic deroofing was done, and the cyst was removed using an endobag and sent for histopathological examination.

The aspirated fluid was sent for microbiological examination, which demonstrated sterile fluid. However, histopathological examination revealed a cyst wall composed of fibrovascular tissue, with attached hepatic parenchyma exhibiting fatty changes and focal area of necrosis, suggesting an infective aetiology. The cyst wall was lined by thin, flattened epithelium. There was no evidence of daughter cysts, scolex, or granulomatous reaction, making the findings suggestive of benign hepatic cyst.

**Figure 2:**
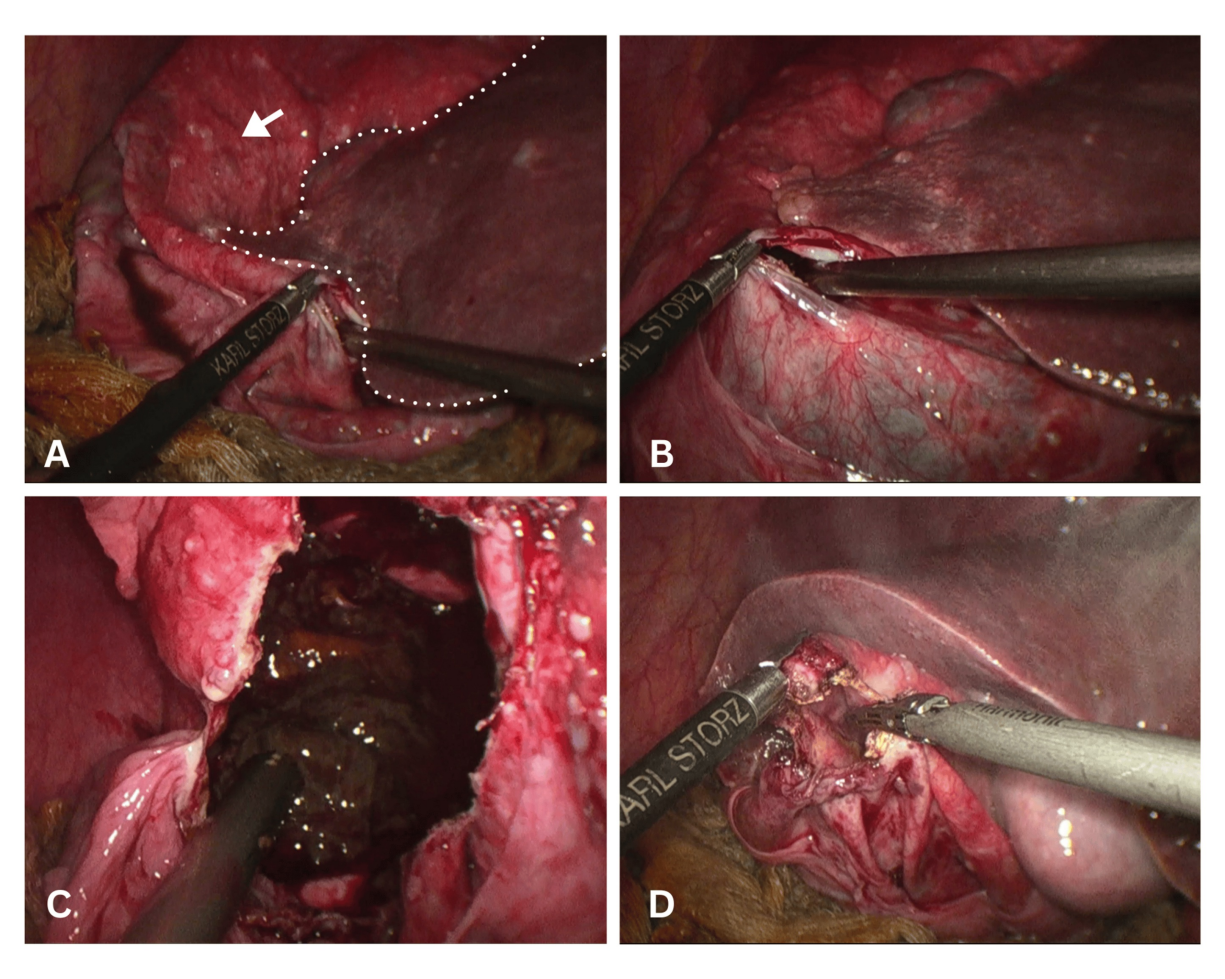
Laparoscopic management of hepatic cyst. (A) Intraoperative laparoscopic view demonstrating the plane between the hepatic cyst wall (white arrow) and the adjacent normal liver parenchyma (dotted line). (B) Controlled decompression of the hepatic cyst using laparoscopic instruments. (C) Opened cyst cavity revealing dirty, infected contents, consistent with an infected hepatic cyst. (D) Deroofing of the cyst, with excision of the cyst wall.

Omentum was tucked at the cyst wall using 2-0 Vicryl sutures. The residual cyst cavity was inspected thoroughly, and hemostasis was achieved. A number 32 drain was placed in the operative field in the subhepatic space. The postoperative period was uneventful, and the patient was started orally on day one and discharged after drain removal on day three. The patient was followed up clinically at one week and two weeks, and with an ultrasound at one month, which showed no findings suggestive of cyst recurrence. The intraoperative findings are shown in Figure 2.

## Discussion

Simple hepatic cysts are benign, non-neoplastic lesions that are lined by simple cuboidal epithelium. They are believed to originate embryologically from aberrant intrahepatic bile ducts that fail to communicate with the biliary tree and undergo progressive dilation over time. These cysts are found more commonly in women, with their incidence increasing with age. Giant cysts are found almost exclusively in people more than 50 years of age [3]. Most cysts follow an indolent course and are discovered incidentally on imaging modalities. An increase in cyst size is sometimes associated with pressure symptoms. The patient typically presents with abdominal distension, vomiting, and pain. Some of the rare presentations of giant hepatic cysts include gastric outlet obstruction, pancreatitis, dyspnea, intestinal obstruction, urinary tract obstruction, and features of right-sided heart failure, all resulting from compression of adjacent organs [2,5,8-11]. Treatment becomes necessary in such cases.

Several treatment modalities have been described, including percutaneous aspiration with sclerotherapy. Although this technique is minimally invasive in nature, it records a high incidence of recurrence, especially in large cysts, as the secretory lining of the cysts is left intact, which leads to accumulation of fluid over time [6]. In such patients, the treatment of choice remains surgical deroofing, which can be done open or through laparoscopy. Traditionally, giant cysts (>10 cm) pose a surgical challenge due to a number of factors, including limited working space and the risk of bleeding and rupture. However, with recent advances in laparoscopic surgery, more and more surgeons are opting for laparoscopic treatment of giant hepatic cysts [7].

In this case report, we presented a case of a giant hepatic cyst (25x20x17 cm) successfully managed laparoscopically. After a search of the PubMed database, we could find only a handful of cases with cyst sizes larger than 20 cm. Table 1 provides a comparative analysis of the cysts reported, along with the treatment modalities used [5,7,12-16]. Compared to non-parasitic cysts, parasitic (hydatid) cysts have been known to achieve much larger dimensions, as reported by Gole et al., of a hydatid cyst measuring 45×35×25 cm [17].

**Table 1:** Size and Treatment Modalities of the Largest Reported Simple Hepatic Cysts in PubMed-Indexed Studies

Literature	Cyst Dimension	Histopathological Examination	Treatment Modality
Bag et al. [5]	25x20x16 cm	No histopathological examination was performed	Ultrasound sonography (USG) guided percutaneous aspiration of fluid
Asuquo et al. [7]	24×20 cm	Reports consistent with simple hepatic cyst	Open cyst deroofing
Mekeel et al. [12]	>25 cm	Reports consistent with simple hepatic cyst	Laparoscopic fenestration
Ikeda et al. [13]	13×17×24 cm	Reports consistent with simple hepatic cyst	Laparoscopic cyst deroofing
Zhang et al. [14]	20.1 cm×12.2 cm×19.6 cm	No histopathological examination was performed	Percutaneous sclerosing therapy [polycinnamol]
Karam et al. [15]	18×23 cm	No histopathological examination was performed	Percutaneous sclerosing therapy (ethanol)
Fischer et al. [16]	22x17x25 cm	No histopathological examination was performed	Laparoscopic fenestration

In our case, we opted for laparoscopic management considering prior failed percutaneous aspiration with recurrence of the cyst and clinical features that were suggestive of superadded infection. Due to these circumstances, definitive surgical management using laparoscopic cyst deroofing was planned. Laparoscopic management of this patient added several benefits over open surgery, including minimal incision size, less postoperative pain, quicker recovery, and early discharge. Benefits over PAS included lower chances of recurrence.

With a measured dimension of approximately 25x20x17 cm and 4 L aspirated intraoperatively, this case is among the larger non-parasitic hepatic cysts reported to be managed laparoscopically.

## Conclusions

Simple hepatic cysts are usually small and asymptomatic and are detected incidentally on imaging modalities. However, some cysts may enlarge sufficiently to cause pressure-related symptoms. Symptomatic hepatic cysts require treatment, which may include percutaneous aspiration with sclerotherapy or surgical deroofing, performed either by open or laparoscopic approach. This report contributes to the literature by demonstrating that laparoscopic management of a large cyst (25x20x17 cm) is also feasible. It suggests that cyst size alone should not be considered a contraindication when deciding between open and laparoscopic approaches.
